# Quantum Mechanical Prediction of Dissociation Constants for Thiazol-2-imine Derivatives

**DOI:** 10.1021/acs.jcim.2c01468

**Published:** 2023-05-01

**Authors:** Evrim Arslan, Zeynep Pinar Haslak, Gérald Monard, Ilknur Dogan, Viktorya Aviyente

**Affiliations:** † Department of Chemistry, Bogazici University, Bebek, 34342 Istanbul, Turkey; ‡ Université de Reims Champagne-Ardenne, 51687 Reims, France; § Université de Lorraine, CNRS, LPCT, F-54000 Nancy, France

## Abstract

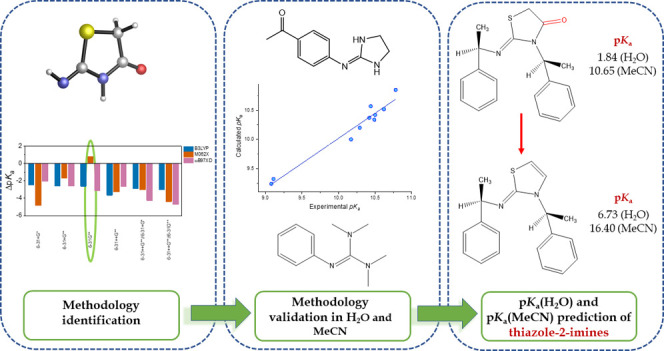

As weak acids or bases, in solution, drug molecules are in either their ionized or nonionized states. A high degree of ionization is essential for good water solubility of a drug molecule and is required for drug–receptor interactions, whereas the nonionized form improves a drug’s lipophilicity, allowing the ligand to cross the cell membrane. The penetration of a drug ligand through cell membranes is mainly governed by the p*K*_a_ of the drug molecule and the membrane environment. In this study, with the aim of predicting the acetonitrile p*K*_a_’s (p*K*_a(MeCN)_) of eight drug-like thiazol-2-imine derivatives, we propose a very accurate and computationally affordable protocol by using several quantum mechanical approaches. Benchmark studies were conducted on a set of training molecules, which were selected from the literature with known p*K*_a(water)_ and p*K*_a(MeCN)_. Highly well-correlated p*K*_a_ values were obtained when the calculations were performed with the isodesmic method at the M062X/6-31G** level of theory in conjunction with SMD solvation model for nitrogen-containing heterocycles. Finally, experimentally unknown p*K*_a(MeCN)_ values of eight thiazol-2-imine structures, which were previously synthesized by some of us, are proposed.

## 1 Introduction

The physicochemical features of a drug molecule, such as solubility, partition coefficient, hydrogen-bonding ability, degree of ionization, and protein binding affinity, are directly related to its therapeutic action. Among these properties, ionization degree (p*K*_a_) plays a significant role in the design of smart drug delivery systems. A high degree of ionization of a drug ligand is a prerequisite for good water solubility and therefore high hydrophilicity, which is required for drug–receptor interactions.

The nonionized form of the ligand improves the lipophilicity of a drug, and therefore, it helps the drug molecule to cross the nonpolar cell membrane.^1−4^ Thus, the penetration of a drug ligand through the cell membranes is mainly governed by the p*K*_a_ of the drug molecule and the membrane environment. On the other hand, since the reactivity and stability of a molecule are highly dependent on its p*K*_a_, the strength of acidity or basicity of a molecule in any solvent determines the mechanisms of chemical reactions involving synthesis, catalysis, oxidation–reduction, and decomposition. In eq 1, the general monoprotic acid (HA) dissociation equilibrium reaction and the deprotonation products (H^+^, A^–^) are shown. The p*K*_a_ of the given equilibrium is the negative logarithm of the equilibrium constant (*K*_a_), as shown in eq 2. The standard free-energy difference between the reactants and products of the dissociation reaction given in eq 1 is related to *K*_a_ and p*K*_a_ as shown in eqs 3 and 4
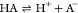
1
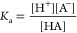
2
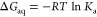
3
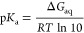
4

**Scheme 1:**
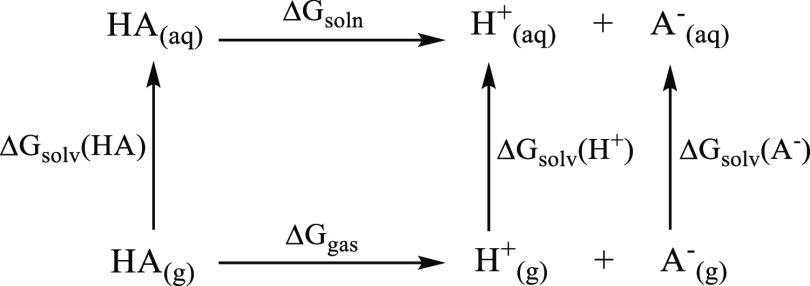
Thermodynamic Cycle 1 (TC1) Where the Acid HA Is Dissociated in Its Conjugate Base A^–^ and a Proton H^+^ Δ*G*_soln_ and Δ*G*_gas_ are the free energies of deprotonation in solution and gas phases, whereas Δ*G*_solv_ is the free energy of solvation.

**Scheme 2:**
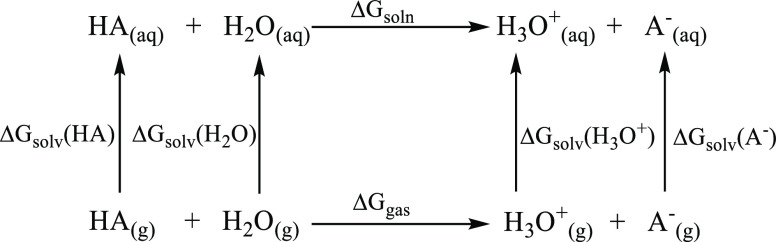
Thermodynamic Cycle 2 (TC2) Where the Acid HA Donates Its Proton H^+^ to the Water Molecule to Yield Its Conjugate Base A^–^ and Hydronium Cation H_3_O^+^ Δ*G*_soln_ and Δ*G*_gas_ are the free energies of deprotonation in solution and gas phases, whereas Δ*G*_solv_ is the free energy of solvation.

Accurate and fast p*K*_a_ predictions by computational methods are crucial for more efficient drug design. Quantum chemical approaches have been extensively used for the prediction of p*K*_a_’s of the ligands.^5−16^ Most of the methods differ in the calculation of the dissociation free energies (Δ*G*_aq_) of a given deprotonation reaction (eq 3). The most straightforward theoretical approach is based on the direct calculation of the Gibbs free energies of the equilibrium reaction of the acid in solution (eq 1). This method, the so-called direct method, faces significant errors arising from the calculation of the free energy of H^+^. In the thermodynamic cycle approaches, TC1 (Scheme 1) and TC2 (Scheme 2), the free energy of solvation, desolvation, and deprotonation of the acid species HA is considered, where Δ*G*_aq_ values can be calculated by
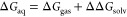
5

Δ*G*_gas_ and ΔΔ*G*_solv_ terms are calculated by ab initio or density functional theory (DFT) methods by adapting the expressions to the thermodynamic cycle constructed. According to the TC1 (Scheme 1), the most commonly implemented thermodynamic cycle, these terms are
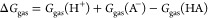
5a
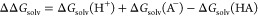
5b

In TC1 Δ*G*_solv_(H^+^), values vary between −265.9 and −270.3 kcal mol^–1^ and this brings large uncertainties to the predicted values.^17^ The large uncertainties coming from the Δ*G*_solv_(H^+^) values in TC1 can be prevented by substituting the proton H^+^ with the H_2_O/H_3_O^+^ pair in TC2 (Scheme 2), in which the Δ*G*_gas_ and ΔΔ*G*_solv_ are calculated as given in eqs 5c and 5d
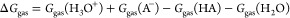
5c
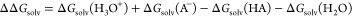
5d

Note that in these reactions the H_2_O is the base and signifies a water cluster, whereas H_(aq)_^+^ and H_3_O_(aq)_^+^ represent the hydrated proton product.^18^ Even though the former is claimed to be simpler and truer than the latter, both models are widely used for p*K*_a_ calculations.

**Scheme 3:**
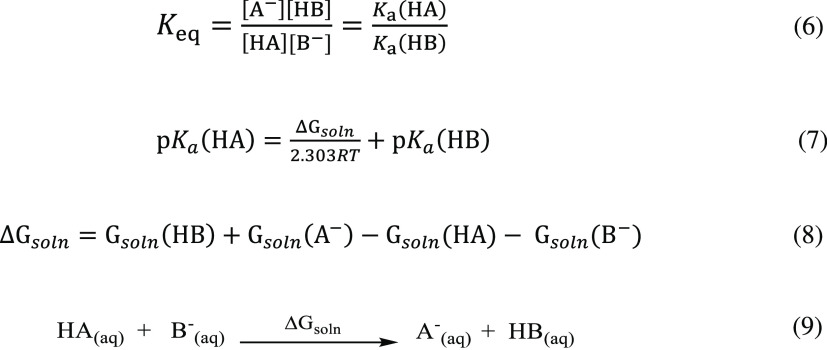
Isodesmic Reaction Scheme Where the Acid HA Donates Its Proton H^+^ to the Base B^–^ to Yield Its Conjugate Base A^–^ and Conjugate Acid HB Δ*G*_soln_ is the free energy of deprotonation in solution.

The isodesmic reaction scheme for p*K*_a_ calculations is based on a reaction between an acid (HA) and the conjugate base of a reference molecule (B^–^) with an experimentally known p*K*_a_ as shown in Scheme 3, whose equilibrium constant is described in eq 6. In the isodesmic reactions, the types of bonds that are made to form the products are the same as those that are broken in the reactant. The p*K*_a_ of acid HA relative to p*K*_a_ of reference molecule HB is calculated by eq 7. Since in the isodesmic method, only the free energies of the reactants and products in solution are calculated, the errors due to the gas-phase calculations are cancelled and as a result more accurate p*K*_a_’s are obtained. The protonation/deprotonation process occurs in similar environments in molecules A and B.

**Figure 1:**
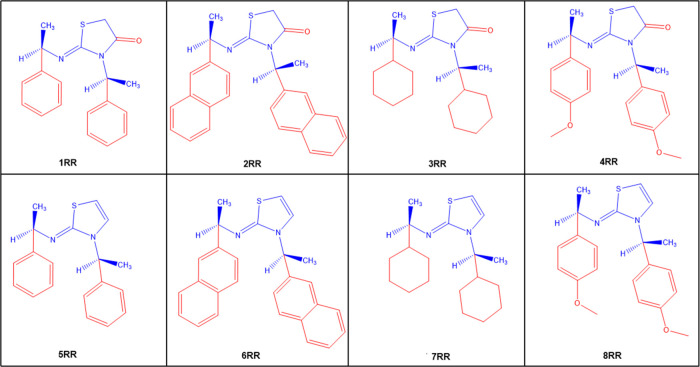
Two-dimensional (2D) representations of the single enantiomer thiazol-2-imines synthesized by Tuncel and Dogan.^26^

**Figure 2:**
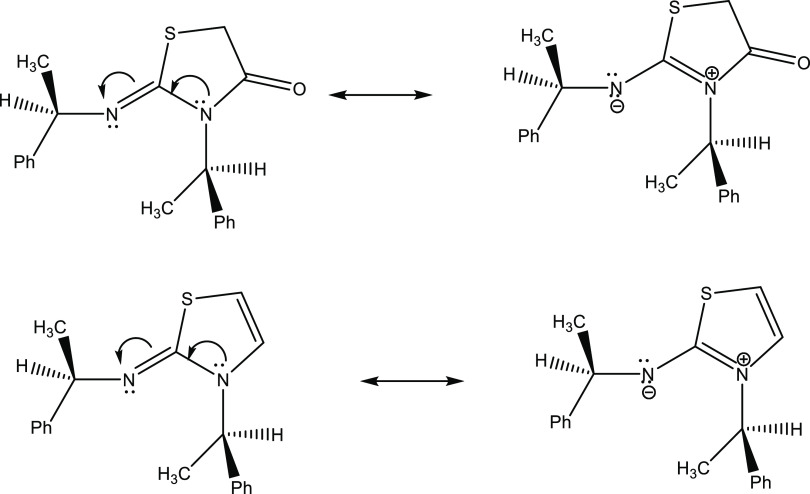
Amidine conjugation in thiazol-2-imine compounds.

With significant biological activities, thiazol-2-imine derivatives are commonly used in organic, synthetic, pharmaceutical, and medicinal chemistry areas due to their anti-inflammatory, analgesic,^19^ antibacterial,^20^ antifungal,^21^ antiviral,^22^ and kinase inhibition activities.^19^ These molecules possess chiral centers; thus, the synthesis of thiazol-2-imine derivatives as single enantiomers is of great interest. Moreover, these thiazol-2-imine derivatives that are present as single enantiomers can be used in asymmetric chemistry such as catalysts in converting olefins to primary amines,^23^ asymmetric hydrogenations,^24^ and generating chiral α-aminonitriles.^25^ Recently, Tuncel and Dogan have synthesized single enantiomer thiazol-2-imines (**5RR**, **6RR**, **7RR**, **8RR**) by water elimination reactions from the corresponding 2-iminothiazolidine-4-ones (**1RR**, **2RR**, **3RR**, **4RR**) (Figure 1).^26^ The synthesized molecules are insoluble in water, and their p*K*_a_’s have been predicted in acetonitrile (MeCN). Due to its relatively lower polarity compared to water, MeCN is a widely used solvent in the pharmaceutical industry since it can mimic the membrane environment.^27,28^ As p*K*_a_ values are strongly dependent on the environment, water p*K*_a_’s may lead to improper conclusions about the degree of ionization of a molecule within the membrane. As an aprotic solvent with a p*K*_a_ of 25, MeCN is a poor H-bond acceptor and a weak base; thus, when it is used as a solvent, it increases the p*K*_a_ values of the solutes, such that acidic compounds act as weaker acids and basic compounds act as stronger bases in MeCN compared to water. Note also that nonaqueous p*K*_a_ data are highly important in many fields since the available experimental data are less abundant than desirable.^29^ Several solvents, including dimethyl sulfoxide, MeCN, and tetrahydrofuran (THF), have found wide application as media for studies of strong bases. MeCN has some advantages over other aprotic solvents: it is a very weakly basic aprotic solvent with a high dielectric constant (36.016) and hence favors the dissociation of ion pairs into free ions. MeCN solvates ions more weakly than water, being a weak electron-pair donor solvates cations better than anions.^4,30^ Compounds **1RR-8RR**, in addition to being drug-like molecules, are expected to be strong chiral organic bases as well because of the amidine conjugation in their structures (Figure 2). Since they are anticipated to be stronger bases than their amine precursors, their p*K*_a_ values are worth determining in different media.

In this study, the acetonitrile and water p*K*_a_’s of thiazol-2-imine derivatives will be predicted by employing the isodesmic reaction scheme based on the previously reported success of the method.^14,31−35^ With this purpose, by using several quantum mechanical approaches, we propose a very accurate protocol. In the first part, the methodology to be employed throughout the study is identified from a benchmark study, where several DFT functionals and solvent models are examined together with TC1 and TC2 schemes, for reproducing the experimentally known water p*K*_a_ of the reference molecule to be used in the isodesmic reaction. In the second and third parts, the proposed methodology is validated on two sets of test molecules whose water and acetonitrile p*K*_a_’s are experimentally known. In the fourth part, p*K*_a(MeCN)_ of the reference molecule to be used in the isodesmic reaction is calculated by the established methodology, and finally, p*K*_a(MeCN)_’s of thiazol-2-imines are calculated by using the isodesmic reaction scheme. The protocol proposed in this study can be used with confidence to predict the water and acetonitrile p*K*_a_’s of thiazol-2-imine derivatives.

## 2 Computational Methodology

A systematic conformational search was conducted for all of the molecules employing the semiempirical PM3 method^36^ by using the SPARTAN14 software.^37^ Free rotations around single bonds were taken into account, and all of the geometries corresponding to stationary points have been reoptimized by using the Gaussian16 program suite.^37^ For the benchmarking study of the DFT method, combinations of a hybrid-GGA exchange–correlation functional (B3LYP),^38^ a hybrid meta-GGA functional (M062X),^39^ and a hybrid-GGA exchange–correlation functional (ωB97XD)^40^ with 6-31+G*, 6-31G**, 6-31+G**, 6-31++G**, and 6-311++G** basis sets were tested. The universal solvent model (SMD)^41^ and the conductor-like polarizable continuum model (CPCM)^42^ were employed to mimic the aqueous solvent environment. The partial atomic charges were derived from Natural Population Analysis (NPA),^43^ Charge Model 5 (CM5),^44^ and Hirshfeld Analysis.^45^ root-mean-square error (RMSE), mean absolute deviation (MAD), and MD (mean deviation) were calculated to compare the efficiency of our predictions.

## 3 Results and Discussion

Since thiazol-2-imines synthesized by Tuncel and Dogan^26^ in Figure 1 are water-insoluble molecules, their p*K*_a_’s are predicted in an acetonitrile environment by employing the isodesmic reaction scheme. With this respect, several quantum chemical approaches were utilized. In the first place, in order to propose a methodology that describes the system best, the experimentally known water p*K*_a_ of the reference molecule to be used in the isodesmic reaction was estimated, employing the TC1 and TC2 schemes with a set of different DFT functionals and solvent models. In the second part of the study, the proposed methodology was validated on a set of 2-(phenylimino)imidazolidine derivatives, and then in the third part the verification of the methodology in an acetonitrile environment was performed for a set of nitrogen-containing heterocyclic compounds. The successful applications of the proposed methodology allowed us to predict the p*K*_a(MeCN)_ of the reference molecule, 2-imino-thiazolidinone. And finally, with the predicted acetonitrile p*K*_a_ of the reference molecule, the p*K*_a(MeCN)_’s of thiazol-2-imines in Figure 1 were calculated by employing the isodesmic reaction scheme.

### 3.1 Identification of the Methodology

**Figure 3:**
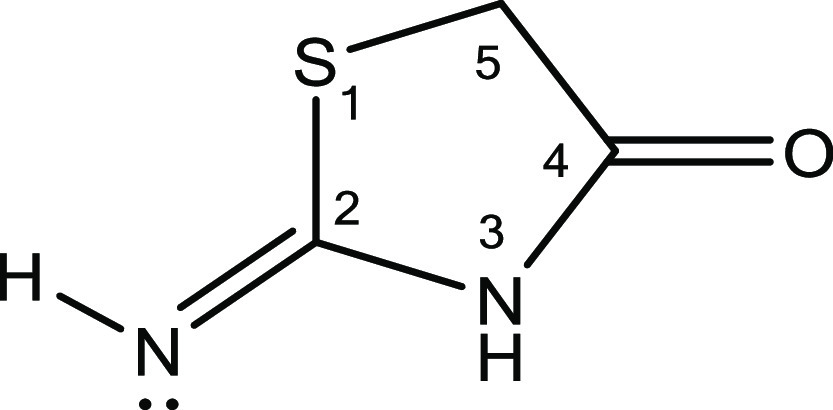
2D representation of the reference molecule 2-imino-thiazolidinone.

As the reference molecule to be used in the isodesmic reaction scheme for the prediction of p*K*_a(MeCN)_’s of thiazol-2-imines, structurally resembling 2-imino-thiazolidinone (Figure 3) with the experimental water p*K*_a_ of 11.70^46^ is used. Since the experimental p*K*_a(MeCN)_ of the reference molecule is not known, a 3-step protocol is followed to predict its p*K*_a(MeCN)_. As well as the calculation scheme to be applied, the accuracy of the prediction is directly related to the quality of the QM method and the solvation model employed to describe the system’s electronic nature and solute–solvent interactions. Therefore, the computational methodology to be used is identified by employing TC1 and TC2 schemes with a set of different DFT functionals, basis sets, and solvation models for the estimation of the water p*K*_a_ of the reference molecule. The reference molecule has two conformers: hydrogen on 2-imine N being either up (toward S) or down (toward N3). “Hydrogen up” conformation, which was calculated to be 0.76 kcal/mol more stable than the “down” conformation, is further considered in our calculations.

As there are two protonation sites in the reference molecule (N3 and 2-imino N), NPA, CM5, and Hirschfeld charge analysis were performed for the identification of the dominant protonation/deprotonation site. The computed charges with all methodologies were found to give the same trend, with 2-imino N having a higher electron density (*q*_N(NPA)_ = −0.628*e*) compared to N3 (*q*_N3(NPA)_ = −0.521*e*). Thus, the reported partial atomic charges of neutral and protonated (on 2-imino N) 2-imino-thiazolidinone are based on NPA calculations in the rest of the manuscript. First, the TC1 scheme displayed in Scheme 1 was employed with several levels of theory in order to predict the water p*K*_a_ of the reference molecule. Two Δ*G*_solv_(H^+^) values (−265.9 kcal/mol and −270.3 kcal/mol) were used for each trial, and the results presented in Table 1 show that the TC1 scheme predicts much lower p*K*_a_’s than that of the experimental value, which indicates the presence of a systematic error. TC1 seems to fail in the prediction of 2-imino-thiazolidinone p*K*_a_ with a very high Δp*K*_a_ (the difference between the predicted and experimental p*K*_a_) range (2.56 ≤ |Δp*K*_a_| ≤ 10.43) irrespective of the level of theory used. When Δ*G*_solv_(H^+^) is taken as −270.3 kcal/mol, the calculated 2-imino-thiazolidinone p*K*_a_ values (calculated p*K*_a_^2^) with respect to different levels of theory are observed to deviate more from the experimental value compared to calculations performed with Δ*G*_solv_(H^+^) = −265.9 kcal/mol (calculated p*K*_a_^1^).

**Table 1:** Benchmark Study for the Prediction of Water p*K*_a_ of the Reference Molecule 2-Imino-thiazolidinone (Experimental p*K*_a_ = 11.70) by Employing the TC1 Scheme^a^^,^^b^

level of theory	calculated p*K*_a_^1^	Δp*K*_a_^1^	calculated p*K*_a_^2^	Δp*K*_a_^2^
B3LYP/6-31+G* CPCM	2.13	–9.57	0.27	–10.43
B3LYP/6-31+G** CPCM	2.45	–9.25	0.59	–10.11
B3LYP/6-31G** CPCM	7.68	–4.02	5.82	–5.88
B3LYP/6-31++G** CPCM	3.65	–8.05	1.79	–9.91
B3LYP/6-31++G**//B3LYP/6-31+G* CPCM	3.63	–8.07	1.77	–9.93
B3LYP/6-31++G**//B3LYP6-31G** CPCM	3.62	–8.08	1.76	–9.94
B3LYP/6-31+G* SMD	6.55	–5.15	4.68	–7.02
B3LYP/6-31+G** SMD	6.60	–5.10	4.74	–6.96
B3LYP/6-31G** SMD	5.57	–5.13	3.83	–7.87
B3LYP/6-31++G** SMD	6.67	–5.03	4.40	–7.30
B3LYP/6-31++G**//B3LYP/6-31+G* SMD	6.02	–5.68	3.90	–7.80
B3LYP/6-31++G**//B3LYP6-31G** SMD	6.14	–5.56	4.48	–7.22
ωB97XD/6-31+G* CPCM	3.75	–7.95	1.89	–9.81
ωB97XD/6-31+G** CPCM	5.62	–6.08	4.70	–7.00
ωB97XD/6-31G** CPCM	8.49	–3.21	6.63	–5.07
ωB97XD/6-31++G** CPCM	5.15	–6.55	3.29	–8.41
ωB97XD/6-31++G**//ωB97XD/6-31+G* CPCM	5.14	–6.56	3.28	–8.42
ωB97XD/6-31++G**//ωB97XD/6-31G** CPCM	5.15	–6.55	2.29	–9.41
ωB97XD/6-31+G* SMD	7.59	–4.11	6.02	–5.68
ωB97XD/6-31+G** SMD	8.00	–3.70	6.14	–5.56
ωB97XD/6-31G** SMD	7.43	–4.27	5.49	–6.21
ωB97XD/6-31++G** SMD	8.45	–3.25	6.72	–4.98
ωB97XD/6-31++G**//ωB97XD/6-31+G* SMD	6.43	–5.27	4.58	–7.12
ωB97XD/6-31++G**//ωB97XD/6-31G** SMD	7.00	–4.70	5.92	–5.78
M062X/6-31+G* CPCM	5.55	–6.15	4.68	–7.02
M062X/6-31+G** CPCM	5.69	–6.01	4.23	–7.47
M062X/6-31G** CPCM	8.01	–3.69	6.75	–4.95
M062X/6-31++G** CPCM	5.59	–6.11	4.02	–7.68
M062X/6-31++G**//M062X/6-31+G* CPCM	5.52	–6.18	3.69	–8.01
M062X/6-31++G**//M062X/6-31G** CPCM	5.01	–6.69	3.53	–8.17
M062X/6-31+G* SMD	4.59	–7.11	2.73	–8.97
M062X/6-31+G** SMD	4.70	–7.00	2.84	–8.86
M062X/6-31G** SMD	9.14	–2.56	7.28	–4.42
M062X/6-31++G** SMD	6.04	–5.66	4.18	–7.52
M062X/6-31++G**//M062X/6-31+G* SMD	6.01	–5.69	4.15	–7.55
M062X/6-31++G**//M062X/6-31G** SMD	5.95	–5.75	4.09	–7.61

a Calculated p*K*_a_^1^: Δ*G*_solv_ (H^+^) value is taken as −265.9 kcal/mol.

b Calculated p*K*_a_^2^: Δ*G*_solv_ (H^+^) value is taken as −270.3 kcal/mol.

**Figure 4:**
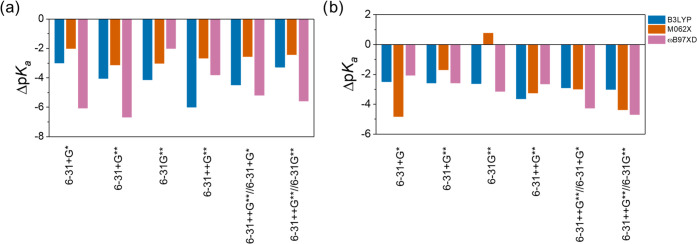
Differences between the predicted and experimental p*K*_a_ (Δp*K*_a_) for three different DFT functionals and six different basis sets considered in this study. Geometry optimizations were performed using the (a) CPCM model and (b) SMD model.

As an attempt to prevent the large uncertainties arising from the Δ*G*_solv_(H^+^) values in TC1, the proton H^+^ was substituted by the H_2_O/H_3_O^+^ pair in TC2 (Scheme 2), and the calculated water p*K*_a_ values of 2-imino-thiazolidinone with different levels of theory are presented in Table 2. The differences between the predicted and experimental p*K*_a_ (Δp*K*_a_) have been calculated for each level of theory and are represented in Figure 4. In the TC2 scheme, a lower Δp*K*_a_ range (0.77 ≤ |Δp*K*_a_| ≤ 6.06) is observed compared to the TC1 scheme. As seen from Figure 4, with the CPCM solvation model, the M062X functional behaves better than the others except for the 6-31G** basis set; on the other hand, with the SMD solvation model, the 6-31G** basis set where polarization function yields the smallest Δp*K*_a_ value.

**Table 2:** Benchmark Study for the Prediction of Water p*K*_a_ of the Reference Molecule 2-Imino-thiazolidinone (Experimental p*K*_a_ = 11.70) by Employing the TC2 Scheme

level of theory	calculated p*K*_a_	Δp*K*_a_
B3LYP/6-31+G* CPCM	8.17	–3.00
B3LYP/6-31+G** CPCM	7.65	–4.05
B3LYP/6-31G** CPCM	7.55	–4.15
B3LYP/6-31++G** CPCM	5.69	–6.01
B3LYP/6-31++G**//B3LYP/6-31+G* CPCM	7.20	–4.50
B3LYP/6-31++G**//B3LYP/6-31G** CPCM	8.42	–3.28
B3LYP/6-31+G* SMD	9.21	–2.49
B3LYP/6-31+G** SMD	9.12	–2.58
B3LYP/6-31G** SMD	9.08	–2.62
B3LYP/6-31++G** SMD	8.05	–3.65
B3LYP/6-31++G**//B3LYP/6-31+G* SMD	7.80	–2.90
B3LYP/6-31++G**//B3LYP/6-31G** SMD	8.69	–3.01
ωB97XD/6-31+G* CPCM	5.64	–6.06
ωB97XD/6-31+G** CPCM	5.01	–6.69
ωB97XD/6-31G** CPCM	9.68	–2.02
ωB97XD/6-31++G** CPCM	7.90	–3.80
ωB97XD/6-31++G**//ωB97XD/6-31+G* CPCM	6.50	–5.20
ωB97XD/6-31++G**//ωB97XD/6-31G** CPCM	6.12	–5.58
ωB97XD/6-31+G* SMD	9.64	–2.06
ωB97XD/6-31+G** SMD	9.12	–2.58
ωB97XD/6-31G** SMD	8.55	–3.15
ωB97XD/6-31++G** SMD	9.05	–2.65
ωB97XD/6-31++G**//ωB97XD/6-31+G* SMD	7.43	–4.27
ωB97XD/6-31++G**//ωB97XD/6-31G** SMD	7.01	–4.69
M062X/6-31+G* CPCM	9.68	–2.02
M062X/6-31+G** CPCM	8.56	–3.14
M062X/6-31G** CPCM	7.98	–3.02
M062X/6-31++G** CPCM	9.02	–2.68
M062X/6-31++G**//M062X/6-31+G* CPCM	9.13	–2.57
M062X/6-31++G**//M062X/6-31G** CPCM	9.27	–2.43
M062X/6-31+G* SMD	6.87	–4.83
M062X/6-31+G** SMD	10.00	–1.70
M062X/6-31G** SMD	12.47	0.77
M062X/6-31++G** SMD	8.45	–3.25
M062X/6-31++G**//M062X/6-31+G* SMD	8.70	–3.00
M062X/6-31++G**//M062X/6-31G** SMD	7.32	–4.38

When the M062X/6-31G** level of theory is used in the presence of the SMD solvation model, the calculated p*K*_a_ of 2-imino-thiazolidinone by employing the TC2 scheme is 12.47, for which the |Δp*K*_a_| is 0.77 unit. Therefore, in order to predict the p*K*_a(MeCN)_ of the reference molecule, further verification of the established methodology is performed on a set of test molecules.

### 3.2 Validation of the Methodology for Water Environment

**Figure 5:**
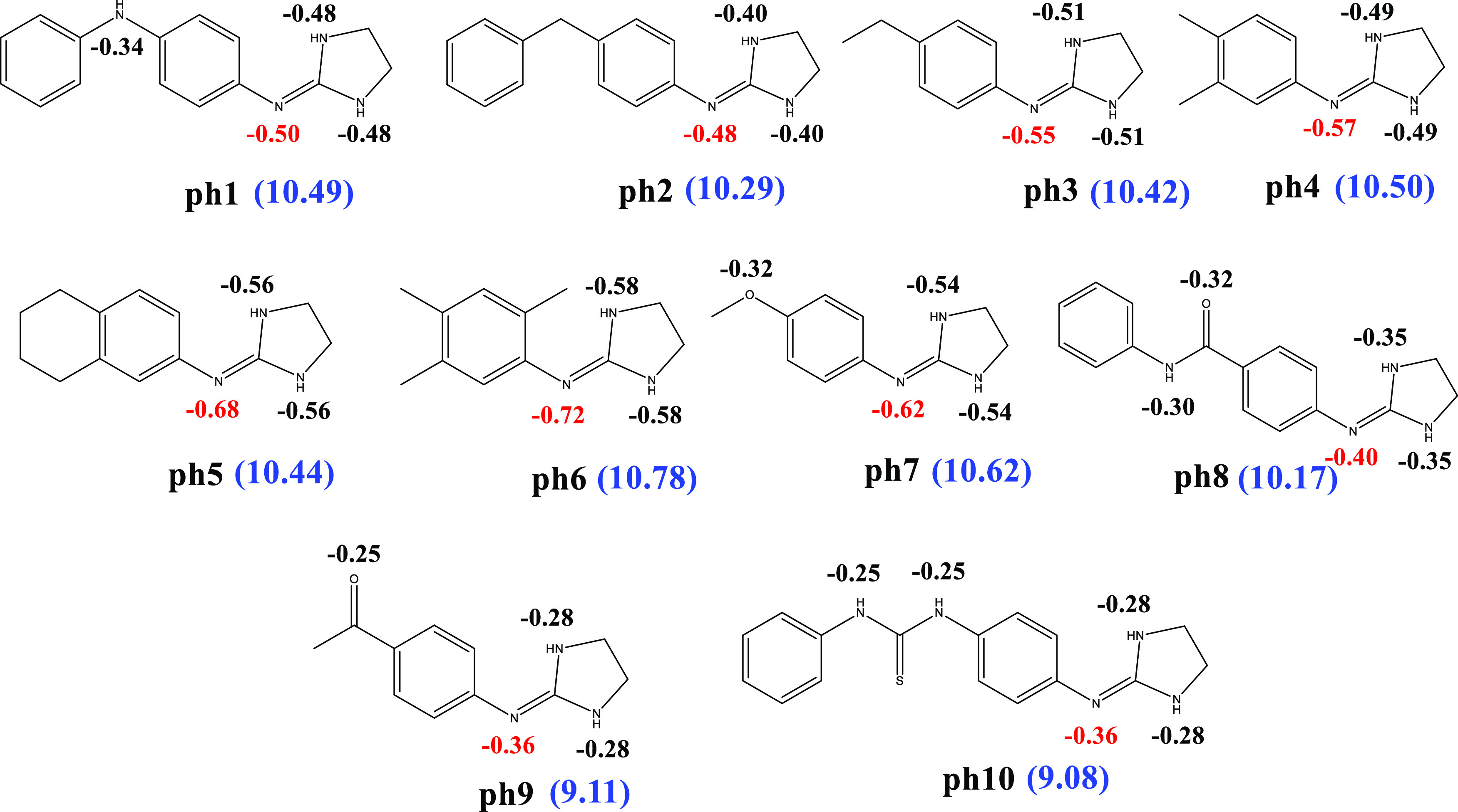
2D representations of 2-(phenylimino)imidazolidine derivatives with computed NPA charges on the heteroatoms (the most negative one is colored with red) (M062X/6-31G**//SMD = water, pop = npa) (experimental p*K*_a_’s are given in blue).

For the validation of the methodology established, a set of molecules with experimentally known water p*K*_a_ were collected from the literature. The selected 10 2-(phenylimino)-imidazolidine derivatives share common scaffolds with our target molecules, thiazol-2-imines, as displayed in Figures 5 and S1, and the p*K*_a_’s of these molecules vary between 9.08 and 10.78.^47^ The methodology proposed in the previous section is applied to the molecules in Figure 5 in order to predict their p*K*_a_’s in water. Initially, NPA charges on the N atoms were calculated for all of the structures and the most electron-rich N atom was selected as the first protonation site. For each molecule, the electron density around imine N was observed to be the highest and that their p*K*_a_’s are directly related to the electron density around the N atom. For example, **ph6** has the highest p*K*_a_ value (10.78) and the computed NPA charge on imine N for **ph6** is the lowest (*q*_N_ = −0.781*e*), whereas **ph10** has the lowest p*K*_a_ (9.08) with the highest NPA charge (*q*_N_ = −0.362*e*) among the **ph** molecules. The presence of electron-withdrawing groups such as carbonyl moieties in the structures of **ph8**, **ph9**, and **ph10** makes the imine N more electron-deficient compared to **ph1**–**7** and as a result less basic. To estimate the water p*K*_a_’s of the 10 molecules displayed in Figure 5, the TC2 scheme with the M062X/6-31G** level of theory in conjunction with the SMD solvation model using water as a solvent was applied by protonating the imine N.

**Figure 6:**
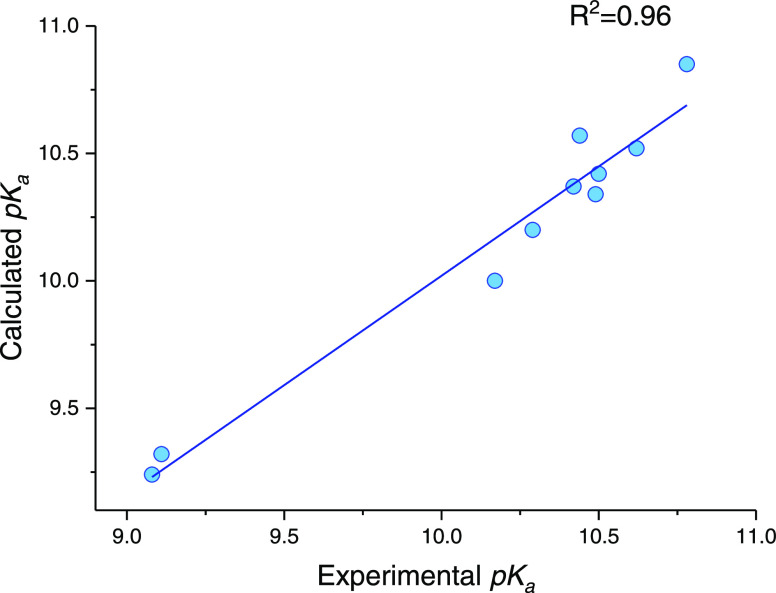
Linear regression of experimental vs calculated p*K*_a_ values of 2-(phenylimino)-imidazolidine derivatives (M062X/6-31G**/SMD = water).

The results presented in Table S1 show that the predicted p*K*_a_’s of 2-(phenylimino) imidazolidine derivatives are very close to their experimentally determined values with a strong correlation (*R*^2^ = 0.96; Figure 6). This impacts the success of the proposed methodology with a high accuracy of MAD and Δp*K*_a_ values (MAD = 0.12 and 0.05 ≤ |Δp*K*_a_ | ≤ 0.21). We additionally assessed the prediction power of our method by comparing the assigned p*K*_a_’s on imine N of 2-(phenylimino) imidazolidine derivatives with ChemAxon,^48^ which is a commercial p*K*_a_ prediction tool widely used in drug development processes. ChemAxon uses the empirically calculated partial atomic charges in molecules as parameters to identify the micro p*K*_a_’s. The predicted p*K*_a_’s by ChemAxon listed in Table S1 are mostly found to be in good agreement with the experimental values except **ph8** and **ph9**, which deviate more than 1 unit. However, the correlation between the predicted and experimental p*K*_a_’s are observed to be weaker (*R*^2^ = 0.31; Figure S2) with a higher MAD value (0.57) compared to the methodology developed in this study.

### 3.3 Validation of the Methodology for Acetonitrile Environment

**Figure 7:**
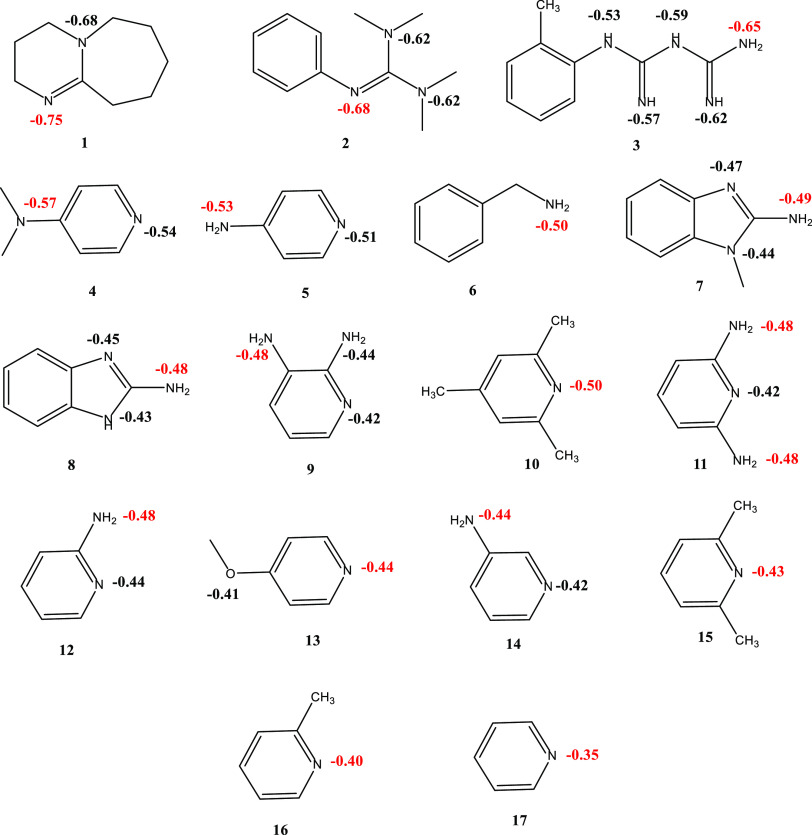
2D representations of selected nitrogen-containing heterocycles with NPA charges on the heteroatoms (the most negative one is colored with red) (M062X/6-31G**/SMD = MeCN, pop = NPA).

Following the validation of the proposed methodology in water environment, the applicability of the methodology was tested for a set of molecules for which there are experimentally determined p*K*_a(MeCN)_’s. The selected 17 molecules are nitrogen-containing heterocycles as displayed in Figures 7 and S3. Since some of the molecules possess more than one N atom in their structures, calculated NPA atomic charges on the N atoms allowed us to determine the most electron-rich N to identify the first protonation site. For the molecules that possess a -NH_2_ functional group (**3**, **5**, **6**, **7**, **8**, **9**, **11**, **12**, **14**), the highest electron density is observed to be located on the N atom of the amino substituent. Then, imine N and N on the -N(CH_3_)_2_ substituent were calculated to have more electron density in molecules **2** and **4**, respectively. The lack of amidine conjugation in **10**, **13**, **15**, **16**, and **17** makes these molecules less basic compared to that of **1**, as predicted by the calculated partial charges on N atoms. TC2 scheme was employed with the M062X/6-31G** level of theory in conjunction with the SMD solvation model using acetonitrile as a solvent for the prediction of p*K*_a(MeCN)_’s of nitrogen-containing heterocycles.

The experimental and predicted p*K*_a_’s of N-containing aromatic compounds are presented in Table 3. The maximum deviation of the predicted p*K*_a(MeCN)_’s from experimental p*K*_a(MeCN)_’s is found to be 0.77 unit, and a very good agreement between calculated and experimental values was obtained with *R*^2^ = 0.98 (Figure S4). Moreover, a small MAD value calculated (0.33) demonstrates the applicability of the proposed methodology to the acetonitrile environment. In addition to the p*K*_a(MeCN)_ calculations, the water p*K*_a_’s of the molecules in Figure 7 are calculated with the proposed methodology and the results are presented in Table 3. Among the 17 selected molecules, experimental p*K*_a(water)_’s were collected from the literature for 14 of them. A very good correlation is obtained between the calculated and the experimental p*K*_a_’s with a maximum deviation of 0.53 units. MeCN is a polar aprotic solvent with a polarized C≡N triple bond. Unlike water, it does not donate hydrogens to anions, and therefore, when used as a solvent, the ionization of the molecules in MeCN is more difficult compared to that of water, which in turn results in higher p*K*_a_’s and thus stronger basicity’s in MeCN environment. The p*K*_a_ for these molecules in MeCN ranges between 24.77 and 12.56, whereas in water it is between 13.50 and 5.23.^30^

**Table 3:** Calculated and Experimental p*K*_a_’s of Nitrogen-Containing Small Aromatic Compounds Displayed in Figure 7 (M062X/6-31G**/SMD = MeCN/Water)

ID	experimental p*K*_a_ (MeCN)	calculated p*K*_a_ (MeCN)	Δp*K*_a_ (MeCN)	experimental p*K*_a_ (water)	calculated p*K*_a_ (water)	Δp*K*_a_ (water)
**1**	24.77^30^	24.13	–0.64	13.50^49^	13.02	–0.48
**2**	19.83^30^	20.60	0.77	NA	11.56	
**3**	19.36^30^	19.43	0.07	NA	11.30	
**4**	18.11^30^	17.74	–0.37	9.60^50,51^	9.07	–0.53
**5**	17.78^30^	17.40	–0.38	9.17^52^	9.02	–0.15
**6**	16.44^30^	16.70	0.26	9.34^53^	9.06	–0.28
**7**	16.02^30^	16.11	0.09	NA	8.76	
**8**	15.96^30^	15.87	–0.09	NA	8.45	
**9**	14.32^30^	15.03	–0.71	6.78^50^	6.43	–0.35
**10**	15.08^30^	14.77	–0.31	7.43^51^	7.70	0.27
**11**	13.94^30^	14.56	0.12	6.13^50^	5.85	–0.28
**12**	13.88^30^	14.26	0.38	6.82^50^	6.60	–0.22
**13**	14.02^30^	14.04	0.02	6.58^50^	7.00	0.42
**14**	14.31^30^	13.96	–0.35	6.04^50^	5.64	–0.40
**15**	13.77^30^	13.92	0.15	6.72^50^	6.32	–0.40
**16**	12.98^30^	13.11	0.13	5.81^54^	5.19	–0.38
**17**	12.56^30^	12.33	–0.23	5.23^55^	5.43	0.20
**RMSE**			0.39			0.27
**MAD**			0.33			0.21
**MD**			–0.05			–0.08

### 3.4 Prediction of p*K*_a(MeCN)_’s of Thiazol-2-imines

**Scheme 4:**
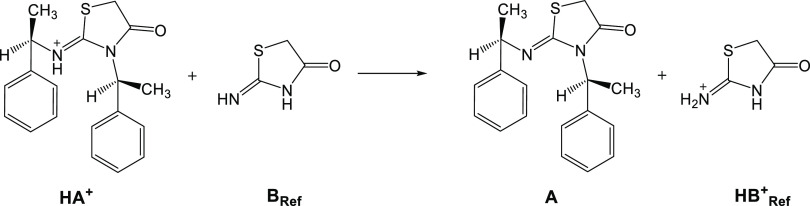
Isodesmic Reaction between an Acid Species (**HA^+^**) and a Reference Species (**B_Ref_**)

In this study, the goal is to predict the p*K*_a_’s of thiazol-2-imines synthesized by Tuncel and Dogan^26^ in an acetonitrile environment. An accurate protocol is suggested by a two-step methodology validation. In the final step of the study, p*K*_a(MeCN)_’s of thiazol-2-imines were calculated by employing the isodesmic reaction scheme at the M062X/6-31G** level of theory in conjunction with the SMD solvation model using acetonitrile as a solvent. Scheme 4 illustrates an isodesmic reaction where the protonated **1RR** is the acidic species **HA**^**+**^ and 2-imino-thiazolidinone is the reference species **B_Ref_**.

To the best of our knowledge, the p*K*_a(MeCN)_ of the reference molecule 2-imino-thiazolidinone is not known experimentally; its p*K*_a(MeCN)_ is first predicted by employing the established methodology. The calculated p*K*_a(MeCN)_ of 2-imino-thiazolidinone using acetonitrile as a solvent is 19.41 (TC2/M062X/6-31G**/SMD), and this prediction will further be used in the next step to propose p*K*_a(MeCN)_’s of thiazol-2-imine derivatives by employing the isodesmic reaction scheme.

The computed charges indicate that the protonation dominantly occurs on the imine N as presented in Table 4 and the predicted p*K*_a_’s of thiazol-2-imines are given in Table 5. We observe that **1RR**, **2RR**, **3RR**, and **4RR** have relatively lower p*K*_a_ values compared to **5RR**, **6RR**, **7RR**, and **8RR**. Withdrawal of the electrons from the exocyclic imine N by the carbonyl substituents on the thiazole ring in **1RR**, **2RR**, **3RR**, and **4RR** results in less electron density around the N atom than the imine N atom of **5RR**, **6RR**, **7RR**, and **8RR** (*q*_N_ = −0.582*e* for **1RR**; *q*_N_ = −0.745*e* for **5RR**) (Table 4). The lower electron density on the protonation site makes these molecules less available for proton abstraction. Thus, **1RR**, **2RR**, **3RR**, and **4RR** were predicted to be less basic compared to **5RR**, **6RR**, **7RR**, and **8RR**. For the molecules of our interest, the p*K*_a_ range in MeCN is between 17.16–10.59 and 7.42–1.77 in water.

**Table 4:** Computed NPA^a^, CM5^b^, and Hirshfeld^c^ Charges of Nitrogen Atoms of Thiazol-2-imines Considered in This Study (M062X/6-31G**/SMD = MeCN)

ID	imine-nitrogen	ring nitrogen
**1RR**	–0.582^a^	–0.336^a^
	–0.616^b^	–0.445^b^
	–0.602^c^	–0.403^c^
**2RR**	–0.594^a^	–0.345^a^
	–0.626^b^	–0.455^b^
	–0.612^c^	–0.413^c^
**3RR**	–0.612^a^	–0.409^a^
	–0.647^b^	–0.489^b^
	–0.625^c^	–0.447^c^
**4RR**	–0.623^a^	–0.421^a^
	–0.659^b^	–0.435^b^
	–0.646^c^	–0.428^c^
**5RR**	–0.745^a^	–0.471^a^
	–0.802^b^	–0.455^b^
	–0.767^c^	–0.463^c^
**6RR**	–0.704^a^	–0.436^a^
	–0.759^b^	–0.448^b^
	–0.738^c^	–0.442^c^
**7RR**	–0.824^a^	–0.503^a^
	–0.865^b^	–0.532^b^
	–0.847^c^	–0.517^c^
**8RR**	–0.805^a^	–0.487^a^
	–0.849^b^	–0.545^b^
	–0.836^c^	–0.503^c^

**Table 5:**
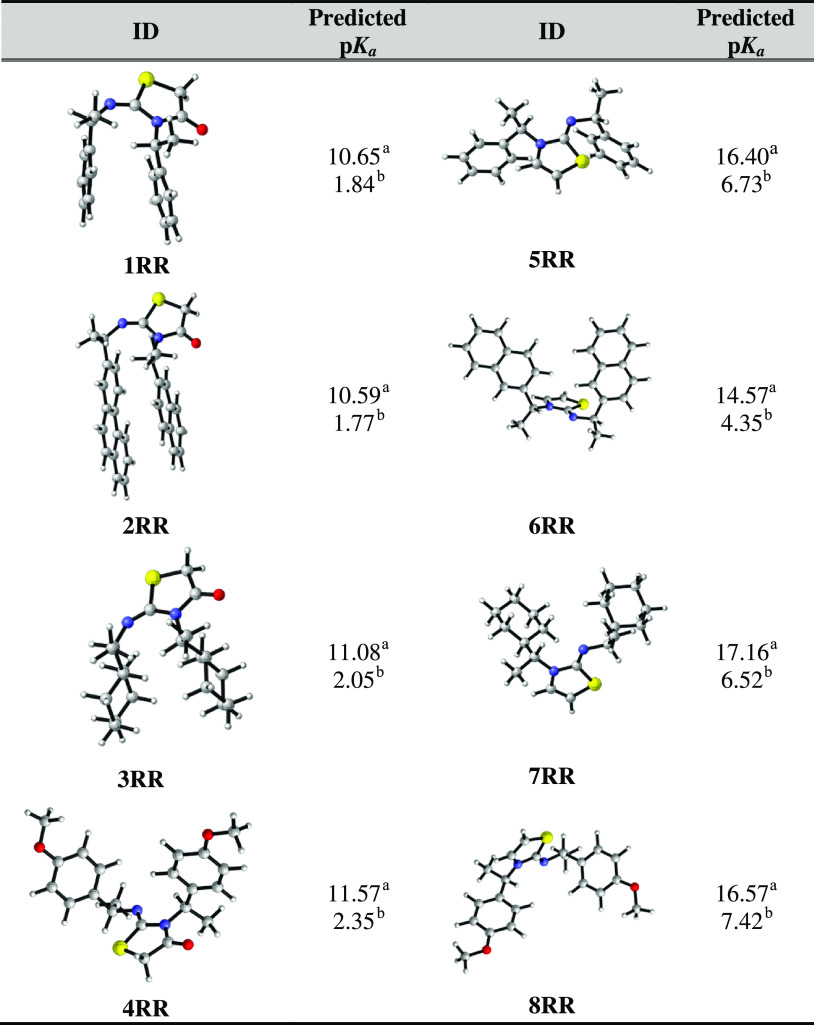
Three-dimensional (3D) Representations of Thiazol-2-imine Derivatives and Their Predicted p*K*_a_’s in MeCN^a^ and Water^b^

### 3.5 Applicability of the Established Methodology to Drug Compounds

We additionally tested our methodology on a small data set (due to the lack of experimental data in the literature) of drug precursors/compounds containing nitrogen heterocycles, which have experimentally determined water and MeCN p*K*_a_’s. Among heterocyclic pharmacophores, the benzimidazole ring system is quite common. Imidazole-containing molecules (benzimidazole, thiabendazole, carbendazim, imidazole) have been chosen since imidazole-based medicinal chemistry suggests the potential therapeutic values of imidazole-derived compounds for treating incurable diseases. Literature survey reveals that the various derivatives of benzimidazole have been synthesized for their pharmacological activities such as antimicrobial,^56^ antifungal,^57^ antiviral,^58^ analgesic,^59^ antiprotozoal,^60^ anticancer,^61^ anti-inflammatory,^62^ antihistaminic,^63^ and antimalarial.^64^ These substructures are often called “privileged” due to their wide recurrence in bioactive compounds.^65^

Besides the imidazole derivatives, we have also evaluated the p*K*_a_ values of guanidine derivatives 1,5,7-triazabicyclo [4.4.0]dec-5-ene (TBD), *N*-methyl TBD (MTBD), and 1-(2-pyridyl)-1,3,4,6,7,8-hexahydro-2*H*-pyrimido[1,2-*a*]pyrimidine (2-(hpp)C_5_H_4_N). Since the Middle Ages in Europe, guanidine has been used to treat diabetes. Due to its long-term hepatotoxicity, further research for blood sugar control was suspended at first after the discovery of insulin. Later development of nontoxic, safe biguanides led to the long-used first-line diabetes control medicine metformin.^66−69^ We attempted to predict the p*K*_a_’s of seven drug compounds by employing the isodesmic scheme at the M062X/6-31G** level of theory with the SMD solvent model in both water and MeCN, and the results presented in Table 6 are found to be in harmony with the experimentally reported p*K*_a_’s. The reference molecule is imidazole for benzimidazole, thiabendazole, and carbendazim; benzimidazole for imidazole. TBD is a reference molecule for MTBD and 2-(hpp)C_5_H_4_N and MTBD for TBD.

**Table 6:**
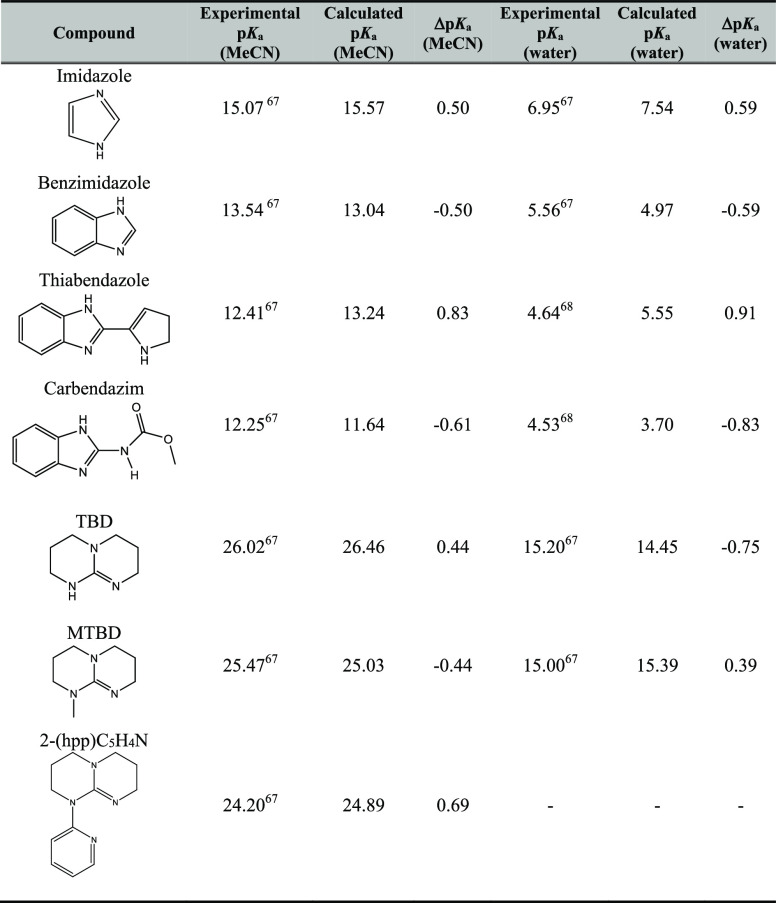
Calculated and Experimental p*K*_a_’s of Drug Precursors/Compounds Containing Nitrogen Heterocycles

## 4 Conclusions

The p*K*_a_ of a drug molecule and the membrane environment are the key factors controlling how well a drug penetrates through the cell membranes. In this study, an elaborate protocol to evaluate the p*K*_a_’s of the single enantiomers of thiazol-2-imines is proposed. Different functionals and basis set combinations were tested to evaluate the p*K*_a(water)_ of a reference molecule resembling the molecules synthesized by Dogan et al.,^26^ 2-imino-thiazolidinone, to be employed in the isodesmic reaction scheme. The M062X/6-31G** level of theory and SMD solvation model are found to be adequate for the prediction of p*K*_a(water)_ of the reference molecule. In order to ensure that the constructed methodology describes the nitrogen-containing heterocyclic compounds well, a two-step validation procedure is followed. First, p*K*_a(water)_ of 2-(phenylimino)imidazolidine derivatives were calculated with the proposed methodology and the predicted values were found to be very close to the experimental p*K*_a(water)_’s. Being interested in p*K*_a_ calculations in a less polar medium, it was necessary to test the methodology for compounds similar to the species of interest whose p*K*_a_ values in acetonitrile are experimentally known. Thus, the applicability of the method was verified on a set of nitrogen-containing heterocyclic compounds with experimentally known p*K*_a(MeCN)_. With the justified methodology, the experimentally unknown p*K*_a(MeCN)_ of the reference molecule, 2-imino-thiazolidinone, was calculated, and finally, isodesmic reactions between the reference molecule and thiazol-2-imine derivatives synthesized by Dogan et al. were proposed to evaluate the p*K*_a(MeCN)_’s of the latter. Moreover, we showed that the protocol established in this study is very satisfying and promising in terms of its applicability to more diverse drug data sets for future validations. The predicted p*K*_a_ values of these drug-like molecules will enable the evaluation of their lipophilicity/hydrophilicity at a given pH.

## Supporting Information Available

3D representations of 2-(phenylimino)imidazolidine derivatives; calculated and experimental water p*K*_a_’s of 2-(phenylimino)imidazolidine derivatives; linear regression of experimental vs ChemAxon calculated p*K*_a_ values of 2-(phenylimino)-imidazolidine derivatives; 3D representations of nitrogen-containing small aromatic compounds; and linear regression of experimental vs calculated p*K*_a_ values of nitrogen-containing small aromatic compounds (PDF)
