# Supplementary material for: Quantum Mechanical Prediction of Dissociation Constants for Thiazol-2-imine Derivatives
Source: J Chem Inf Model. 2023 May 1;63(10):2992–3004. doi: 10.1021/acs.jcim.2c01468 (PMC10207282; doi:10.1021/acs.jcim.2c01468)
Supplement: Supplementary file 1 — ci2c01468_si_001.pdf [file ci2c01468_si_001.pdf]

Supporting Information

for

Quantum-Mechanical Prediction of Dissociation Constants for Thiazol-2-imine Derivatives

Evrim Arslan[a,‡], Zeynep Pinar Haslak[a,b,‡], Gérald Monard[c], İlknur Dogan[a]*, Viktorya Aviyente[a*]

[a]Bogazici University, Department of Chemistry, 34342, Bebek, Istanbul

[b]Université de Reims Champagne-Ardenne, Reims, 51687, France

[c]Université de Lorraine, CNRS, LPCT, F-54000 Nancy, France

[‡]These authors contributed equally.

<u>Table of Contents</u>

Figure S1: 3D representations of 2-(phenylimino)imidazolidine derivatives (M062X/6-31G**/SMD=water)……………………………………………………………………S-3

Table S1: Calculated and experimental water pK_a's of 2-(phenylimino)imidazolidine derivatives (M062X/6-31G**/SMD=water)……………..…..………………………………...S-4

Figure S2. Linear regression of experimental vs ChemAxon calculated pK_a values of 2-(phenylimino)-imidazolidine derivatives………………………………………………….S-5

Figure S3. 3D representations of nitrogen containing small aromatic compounds (M062X/6-31G**/SMD=MeCN)……………………………………………….…….………...S-6

Figure S4. Linear regression of experimental vs calculated pK_a values of nitrogen containing small aromatic compounds (M062X/6-31G**/SMD=MeCN)……………………………S-7

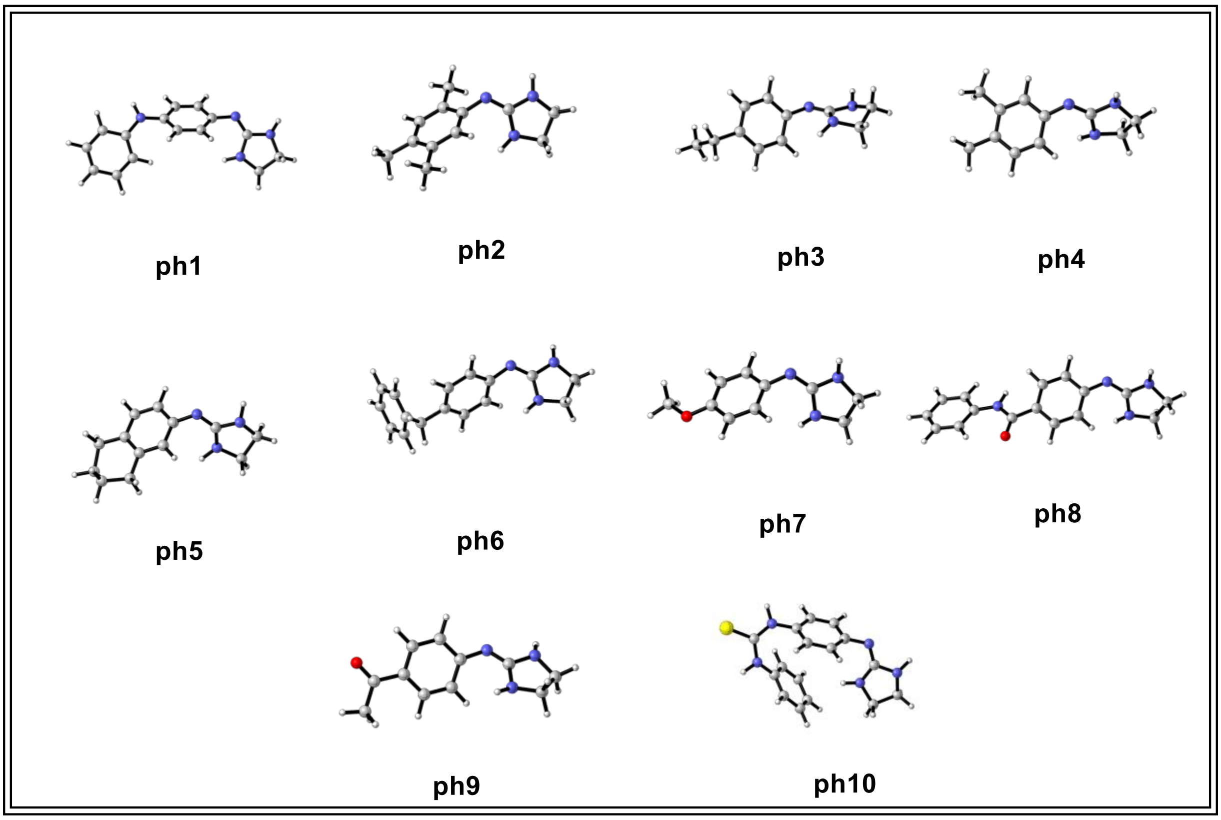

Figure S1. 3D representations of 2-(phenylimino)imidazolidine derivatives (M062X/6-31G**//SMD=water)

Table S1. Experimental pK_a's, calculated pK_a's by employing the TC2 scheme at M062X/6-31G** level of theory with SMD solvation model, differences between experimental and calculated pK_a's (TC2 scheme), calculated pK_a's by ChemAxon, differences between experimental and calculated pK_a's (ChemAxon).

ID	Experimantal pK_a	Calculated pK_a (TC2 Scheme)	ΔpK_a (TC2 Scheme)	Calculated pK_a (ChemAxon)	ΔpK_a (ChemAxon)
**ph1**	10.49	10.34	-0.15	10.10	-0.39
**ph2**	10.29	10.20	-0.09	9.80	-0.49
**ph3**	10.42	10.37	-0.05	11.04	0.62
**ph4**	10.50	10.42	-0.08	11.05	0.55
**ph5**	10.44	10.57	0.13	10.91	0.47
**ph6**	10.78	10.85	0.07	11.11	0.33
**ph7**	10.62	10.52	-0.10	10.96	0.34
**ph8**	10.17	10.00	-0.17	8.97	-1.20
**ph9**	9.11	9.32	0.21	10.32	1.21
**ph10**	9.08	9.24	0.16	9.21	0.13
**RMSE**			0.13		0.67
**MAD**			0.12		0.57
**MD**			-0.01		0.16

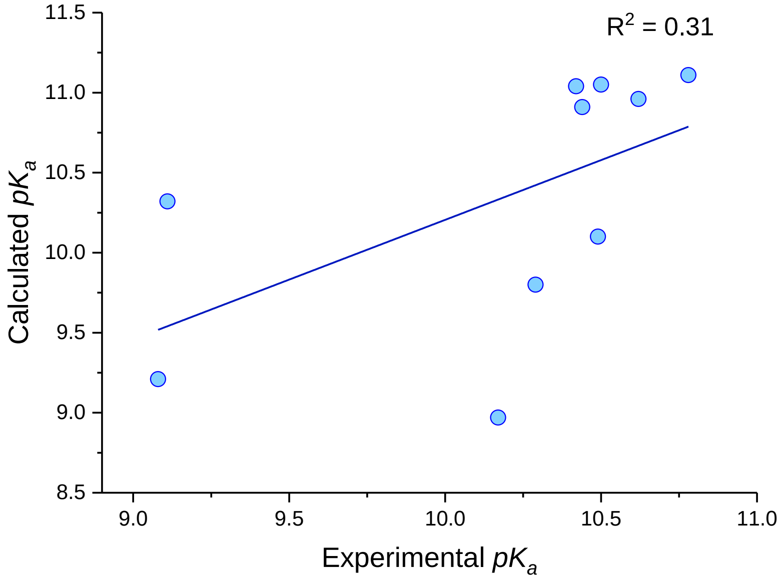

Figure S2. Linear regression of experimental vs ChemAxon calculated pK_a values of 2-(phenylimino)-imidazolidine derivatives.

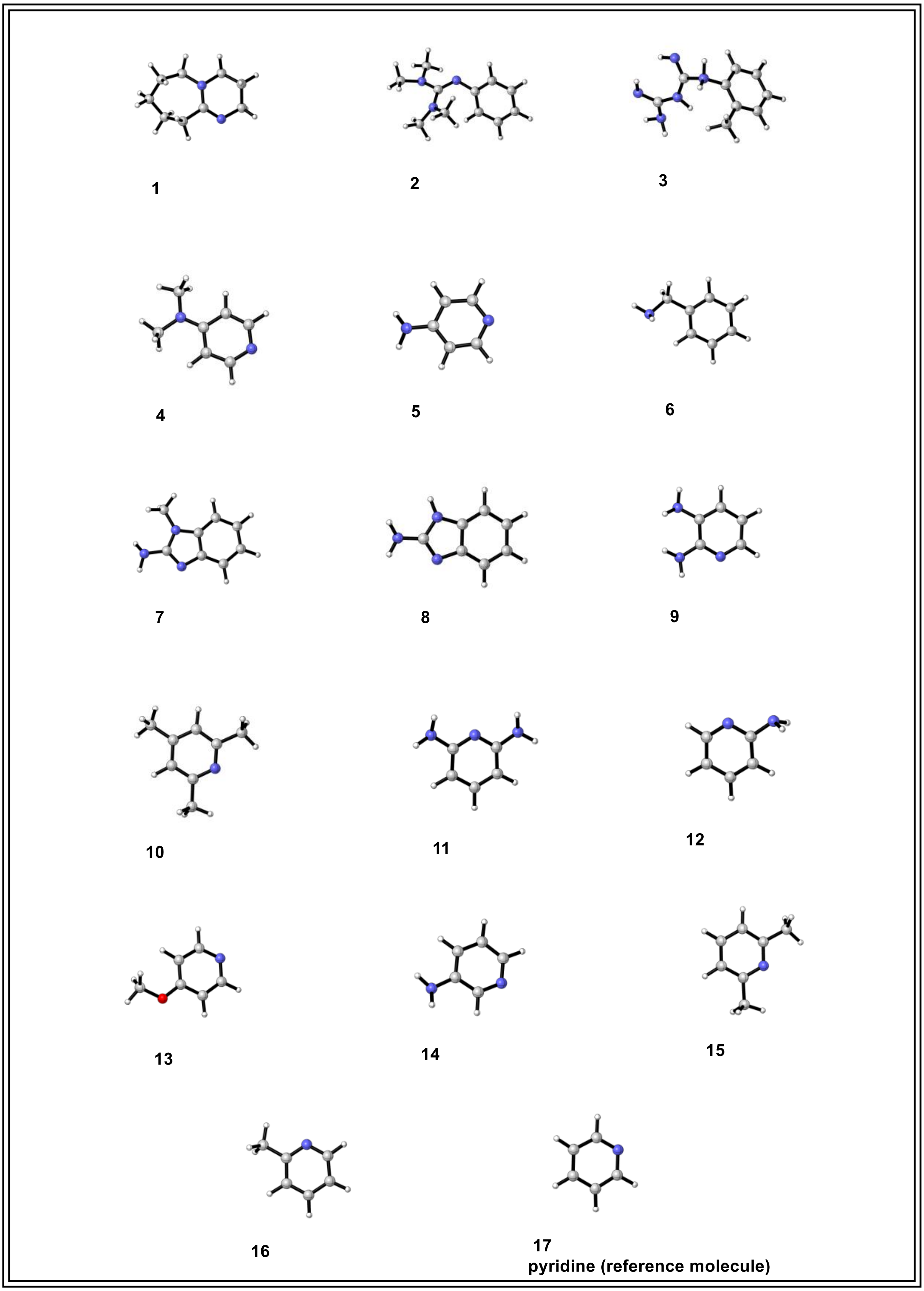

Figure S3. 3D representations of nitrogen containing small aromatic compounds (M062X/6-31G**//SMD=MeCN)

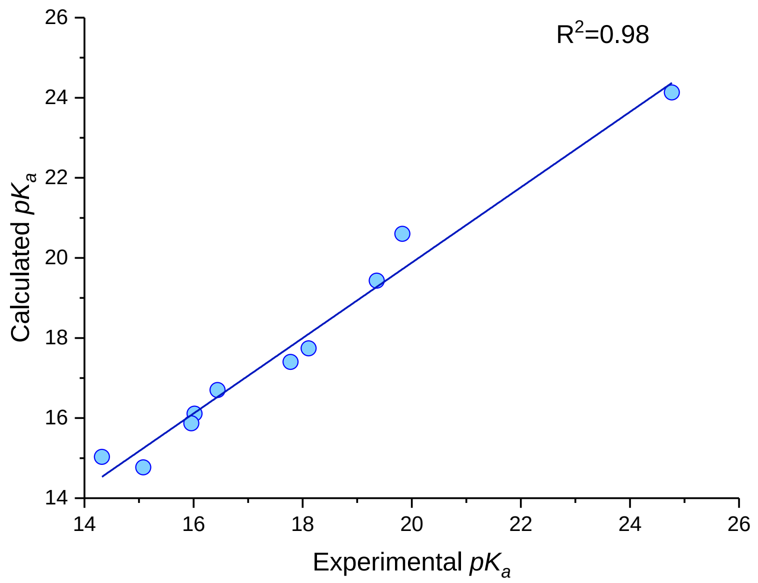

Figure S4. Linear regression of experimental vs calculated pK_a values of nitrogen containing small aromatic compounds (M062X/6-31G**//SMD=MeCN)
